# Co-Infections with Orthomarburgviruses, Paramyxoviruses, and Orthonairoviruses in Egyptian Rousette Bats, Uganda and Sierra Leone

**DOI:** 10.3201/eid3105.241669

**Published:** 2025-05

**Authors:** Brian R. Amman, Amy J. Schuh, Tara K. Sealy, Immah Conteh, Gloria G. Akurut, Alusine H. Koroma, Kilama Kamugisha, James C. Graziano, Emmanuel Saidu, Doris F. Bangura, Emmanuel S. Kamanda, Ibrahim A. Bakarr, Jonathan Johnny, Eric M. Enyel, Jonathan A. Musa, Augustus Osborne, Ibrahim K. Foday, Camilla Bangura, Christian Sumaila, Samuel M.T. Williams, George M. Fefegula, Patrick Atimnedi, Aiah Lebbie, Jonathan S. Towner

**Affiliations:** Author affiliations: Centers for Disease Control and Prevention, Atlanta, Georgia, USA (B.R. Amman, A.J. Schuh, T.K. Sealy, J.C. Graziano, J.S. Towner); Njala University, Bo, Sierra Leone (I. Conteh, A.H. Koroma, E. Saidu, D.F. Bangura, E.S. Kamanda, I.A. Bakarr, J. Johnny, J.A. Musa, A. Osborne, I.K. Foday, C. Bangura, C. Sumaila, S.M.T. Williams, G.M. Fefegula, A. Lebbie); Uganda Wildlife Authority, Kampala, Uganda (G.G. Akarut, K. Kamugisha, E.M. Enyel, P. Atimnedi); University of Sierra Leone, Freetown, Sierra Leone (A. Lebbie)

**Keywords:** Marburg virus, Sosuga virus, Kasokero virus, Yogue virus, Orthomarburgvirus, paramyxoviruses, Orthonairovirus, Egyptian rousette, *Rousettus aegyptiacus*, bat, migration, active infection, co-infection, viruses, zoonoses, Uganda, Sierra Leone

## Abstract

We report 1.3% (19/1,511) of Egyptian rousette bats (ERBs) in Uganda and Sierra Leone were co-infected with different combinations of Marburg, Sosuga, Kasokero, or Yogue viruses. To prevent infection by those viruses, we recommend avoiding ERB-populated areas, avoiding ERBs and ERB-contaminated objects, and thoroughly washing harvested fruits before consumption.

Zoonotic co-infections occur when >2 genetically distinct infectious agents are detected in a single host (*1*) and are common in wildlife (*2*–*4*). Compared with infection by a single pathogen, co-infections can alter host susceptibility to other agents, disease transmission dynamics, and duration of illness (*4*), as well as replication and shedding of infectious agents (*5*).

Bats belong to the second largest order of mammals (order Chiroptera), representing 20% of known mammal species. Bats are also associated with >4,100 distinct animal viruses (*6*). Egyptian rousette bats (ERBs; *Rousettus aegyptiacus*) have been well studied as vertebrate hosts for zoonotic viruses. ERBs are the known reservoir for Marburg virus (MARV) and Ravn virus (family Filoviridae, genus *Orthomarburgvirus*) (*7*), a putative reservoir for Sosuga virus (SOSV; family Paramyxoviridae, genus *Pararubulavirus*) (*8*), and the only known vertebrate reservoir for Kasokero virus (KASV) in East Africa and Yogue virus (YOGV; family Nairoviridae, genus *Orthonairovirus*) in West Africa (*9*). Virus co-infections are also frequently observed in bats; many virus-positive bats in China are co-infected with >2 viruses (*10*). Simultaneous shedding of multiple distinct paramyxoviruses has been reported in flying fox bats (*Pteropus* spp.) in Australia (*11*). Co-infections with multiple rubulavirus-related viruses and other unclassified paramyxoviruses have been reported in ERBs in South Africa (*12*). Virus co-infection in bats has been described as an underappreciated phenomenon in disease ecology and surveillance data reporting (*2*). Here, we investigated zoonotic virus co-infections in ERBs from Uganda and Sierra Leone

## The Study

During filovirus ecology research efforts in Uganda and Sierra Leone, we captured ERBs by using mist nets and harp traps (Table). We collected visceral samples, including liver, spleen, heart, lung, kidney, axillary lymph node, salivary gland, colon, and blood, for virologic testing. We tested the samples for the known zoonotic viruses SOSV, KASV (Uganda; blood samples only), and YOGV (Sierra Leone; blood samples only). We additionally tested samples from 1,511 bats captured during November 2009–February 2022 in Uganda and Sierra Leone (Appendix Table 1) for MARV (nucleoprotein and viral protein 35 genes), SOSV (nucleoprotein, hemagglutinin, and neuraminidase genes) and KASV and YOGV (nucleoprotein genes) by quantitative reverse transcription PCR.

**Table:** Numbers of Egyptian rousette bats (*Rousettus aegyptiacus*) captured according to site in study of co-infections with orthomarburgviruses, paramyxoviruses, and orthonairoviruses, Uganda and Sierra Leone

Captured bats	Uganda					Sierra Leone			Grand total
	Python Cave*	Kasungwa Cave*	Kitaka Mine†	Total no.		Tailu Village‡	Kasewe Cave§	Total no.	
Female	256	53	202	511		2	183	185	696
Adult	140	36	105	281		2	65	67	348
Juvenile	116	17	97	230		0	118	118	348
Male	381	43	197	621		5	189	194	815
Adult	219	41	128	388		2	86	88	476
Juvenile	162	2	69	233		3	103	106	339
Total no.	637	96	399	1,132		7	372	379	1,511

*Stable populations. †Depopulated, undergoing repopulation. ‡Forest caught, population unknown. §Transient population, possible maternal colony.

In ERB samples, we detected individual infections and multiple co-infections with MARV and SOSV (n = 1,132 samples from Uganda; n = 379 from Sierra Leone), KASV (n = 942 from Uganda), and YOGV (n = 275 from Sierra Leone) (Appendix Table 1). For 1,511 bats sampled in both Uganda and Sierra Leone, 6.0% (90/1,511) had detectable MARV RNA and 10.7% (162/1,511) had detectable SOSV RNA; 3.1% (29/942) of ERBs from Uganda only had detectable KASV RNA, and 0.4% (1/275) from Sierra Leone only had detectable YOGV RNA.

In Uganda and Sierra Leone, we detected 23 co-infections with >2 viruses: 10 (43.5%) co-infections in Uganda and 13 (56.5%) in Sierra Leone. Co-infections with MARV and SOSV (1.3% [19/1,511]) were most prevalent in both Uganda (0.8% [9/1,132]) and Sierra Leone (2.6% [10/379]). We did not detect MARV and KASV co-infections in Uganda; we detected only 1 (0.4%) of 275 tested samples co-infected with MARV and YOGV in Sierra Leone. We also detected co-infection with SOSV and KASV in Uganda (0.1% [1/942]) and SOSV and YOGV co-infection in Sierra Leone (0.4% [1/275]). A single ERB from Sierra Leone had detectable RNA for MARV, SOSV, and YOGV (0.4% [1/275]) (Appendix Table 1). 

Among the 19 ERBs co-infected with MARV and SOSV, 9 (47.4%) were female and 10 (52.6%) male. Eight (42.1% [8/19]) of the bats were adults (forearm >89 mm length) and 11 (57.9% [11/19]) were juveniles (forearm <89 mm length).

We detected co-infections with MARV, SOSV, KASV (Uganda only), and YOGV (Sierra Leone only) in ERBs from Uganda and Sierra Leone; most bats were co-infected with MARV and SOSV and primarily at only 1 site in each country. Most MARV/SOSV co-infections in Uganda were observed in bats from the Kitaka Mine sampling during November 2012. The mine was undergoing a resurgence of MARV infections after a depopulation and repopulation event, as previously reported (*13*); MARV active infection rates in ERBs had more than doubled at the mine since 2007 (*7*). The SOSV active infection rates were also much higher at Kitaka Mine than at the undisturbed and permanent ERB colonies at Kasungwa Cave and Python Cave sites (*8*). It is possible that a newly formed naive ERB population occupied the recently re-opened mine, providing an excess of bats not previously exposed to MARV or SOSV. 

A comparable SOSV rate of active infection was identified at Kasewe Cave in Sierra Leone. One possible explanation for high SOSV infections at Kasewe Cave could be that the ERB population there appeared to be migratory, and the cave was mostly devoid of ERBs at certain times of the year (February–April). The ERB population at Kasewe Cave might be an amalgamation of many smaller populations that return annually to the cave site to use area resources and then disperse into smaller populations to return to other, smaller roosting sites. Subsequent reoccupations by smaller ERB populations year after year at Kasewe Cave bring new, virus naive bats together in larger numbers, similar to the repopulation of Kitaka Mine, causing higher numbers of SOSV infections in the population and, subsequently, higher rates of co-infection with MARV. Moreover, the limited data collected at the Kasewe Cave suggest the site could be a maternity roost, but because of that limitation, a definitive designation cannot be made. The ratio of juveniles to adults was higher at Kasewe Cave (59.4% [221/372]) than at Kitaka Mine (41.6% [166/399]), which could also explain the high SOSV prevalence at the cave site given recent findings of an age-related bias in SOSV prevalence at Kasewe Cave (*14*). More surveillance will be needed to determine normal rates of SOSV infection in ERB populations across Sierra Leone.

## Conclusions

It remains unknown why levels of active MARV infection at Kasewe Cave are not following the same repopulation patterns as those at Kitaka Mine or following the rates of SOSV in Kasewe Cave. One possibility involves timing of collection efforts, which might have missed the peak of active MARV infections. Another potential explanation could be that SOSV, and paramyxoviruses in general, are more easily transmitted within ERB populations than MARV, when considering possible SOSV recrudescence and vertical transmission has been seen for other paramyxoviruses, such as Nipah virus (Appendix reference *16*). Increases in rubulavirus-like paramyxovirus transmission during female aggregation and reproduction have been identified in a population of ERBs in South Africa, which showed seasonal population fluctuations similar to those in Sierra Leone (*12*). The high active SOSV infection rate, in addition to the high infection risk associated with MARV co-infection of ERBs in Kasewe Cave, indicates a substantial public health risk because of public harvests of bats for food, as well as guano mining to produce crop fertilizer. SOSV and other rubula-like paramyxoviruses have been reported to be shed in both urine and feces (guano) from infected bats (*12*,*15*). Our public health recommendations to prevent human infection by ERB-borne zoonotic viruses are to avoid entering areas where ERBs are known to roost, avoid contact with ERBs, avoid objects obviously contaminated by ERBs (including guano mining within known ERB roosts), and thoroughly wash all harvested vegetable and fruit produce from those areas before consumption.

Appendix Additional information for co-infections with orthomarburgviruses, paramyxoviruses, and orthonairoviruses in Egyptian rousette bats, Uganda and Sierra Leone.
