# Supplementary material for: Co-Infections with Orthomarburgviruses, Paramyxoviruses, and Orthonairoviruses in Egyptian Rousette Bats, Uganda and Sierra Leone
Source: Emerg Infect Dis. 2025 May;31(5):1015–8. doi: 10.3201/eid3105.241669 (PMC12044226; doi:10.3201/eid3105.241669)
Supplement: Appendix — Additional information for co-infections with orthomarburgviruses, paramyxoviruses, and orthonairoviruses in Egyptian rousette bats, Uganda and Sierra Leone. [file 24-1669-Techapp-s1.pdf]

Article DOI: https://doi.org/10.3201/eid3105.241669

EID cannot ensure accessibility for supplementary materials supplied by authors. Readers who have difficulty accessing supplementary content should contact the authors for assistance.

Co-Infections with Orthomarburgviruses, Paramyxoviruses, and Orthonairoviruses in Egyptian Rousette Bats, Uganda and Sierra Leone

Appendix

Appendix Table 1. Dates of Egyptian rousette bat (*Rousettus aegyptiacus*) captures according to site during pathogen surveillance efforts in Uganda and Sierra Leone*

Location	Capture dates
Uganda	
Python Cave	11/2009, 02/2022
Kasungwa Cave	08/2009
Kitaka Mine	11/2012
Sierra Leone	
Tailu Village	02/2017, 03/2017, 04/2019
Kasewe Cave	10/2017, 11/2017, 09/2018, 10/2018, 11/2018, 11/2019, 10/2020, 11/2020

*Dates are listed by month/year for each roost site.

Appendix Table 2. Marburg, Sosuga, Kasokero, and Yogue virus individual infections and co-infections detected during pathogen discovery surveillance of Egyptian rousette bats (*Rousettus aegyptiacus*) in Uganda and Sierra Leone*

	Uganda				Sierra Leone			
Infected bats	Python Cave	Kasungwa Cave	Kitaka Mine	Total no.	Tailu Village	Kasewe Cave	Total no.	Grand total
No. bat captures	637	96	399	1,132	7	372	379	1,511
Marburg virus	13/637 (2.0)	1/96 (1.0)	53/399 (13.3)	67/1,132 (5.9)	0/7	23/372 (6.2)	23/379 (6.1)	90/511 (6.0)
F	7/249	1/52	25/177	33/478	0/2	16/167	16/169	49
Adult	0/140	0/36	10/95	10/271	0/2	7/58	7/60	17
Juvenile	7/109	1/16	15/82	23/207	0/0	9/109	9/109	32
M	6/375	0/43	28/169	34/587	0/5	7/182	7/187	41
Adult	4/215	0/41	22/106	26/362	0/2	1/85	1/87	27
Juvenile	2/160	0/2	6/63	8/225	0/3	6/97	6/100	14
Sosuga virus	19/637 (3.0)	3/96 (3.1)	41/399 (10.3)	63/1,132 (5.6)	0/7	99/372 (26.6)	99/379 (26.1)	162
F	7/249	3/50	19/183	29/482	0/2	52/131	52/133	66
Adult	6/134	2/34	6/99	14/267	0/2	15/50	15/52	29
Juvenile	1/115	1/16	13/84	15/215	0/0	37/81	37/81	52
M	12/369	0/43	22/175	34/587	0/5	47/142	47/147	47
Adult	6/213	0/41	16/112	22/366	0/2	14/72	14/74	14
Juvenile	6/156	0/2	6/63	12/221	0/3	33/70	33/73	33
Kasokero virus†	24/558 (4.3)	NT	5/384 (1.3)	29/942 (3.1)	NT	NT	NT	29
F	11/208	NT	2/192	13/400	NT	NT	NT	13
Adult	8/118	NT	0/99	8/217	NT	NT	NT	8
Juvenile	3/90	NT	2/93	5/183	NT	NT	NT	5
M	13/326	NT	3/187	16/513	NT	NT	NT	16
Adult	9/200	NT	3/121	12/321	NT	NT	NT	12
Juvenile	4/126	NT	0/66	4/192	NT	NT	NT	4
Yogue virus‡	NT	NT	NT	NT	NT	1/272 (0.4)	1/275 (0.4)	1
F	NT	NT	NT	NT	NT	1/126	1/126	1
Adult	NT	NT	NT	NT	NT	1/46	1/46	1
Juvenile	NT	NT	NT	NT	NT	0/80	0/80	0
M	NT	NT	NT	NT	0/3	0/145	0/148	0
Adult	NT	NT	NT	NT	0/1	0/61	0/62	0
Juvenile	NT	NT	NT	NT	0/2	0/84	0/86	0
Marburg + Sosuga virus	0/637	0/96	9/399 (2.3)	9/1,132 (0.8)	0/7	10/372 (2.7)	10/379 (2.6)	19
F	0/256	0/53	2/200	2/509	0/2	7/176	7/178	9
Adult	0/140	0/36	1/104	1/280	0/2	2/63	2/65	3
Juvenile	0/116	0/17	1/96	1/229	0/0	5/113	5/113	6
M	0/381	0/43	7/190	7/611	0/5	3/186	3/191	10
Adult	0/219	0/41	4/124	4/384	0/2	1/85	1/87	5
Juvenile	0/162	0/2	3/66	3/228	0/3	2/101	2/104	5
Marburg + Kasokero†	0/558	NT	0/384	0/942	NT	NT	NT	0
F	0/219	NT	0/194	0/413	NT	NT	NT	0
Adult	0/126	NT	0/99	0/225	NT	NT	NT	0
Juvenile	0/93	NT	0/95	0/188	NT	NT	NT	0
M	0/339	NT	0/190	0/529	NT	NT	NT	0
Adult	0/209	NT	0/124	0/333	NT	NT	NT	0
Juvenile	0/130	NT	0/66	0/196	NT	NT	NT	0
Marburg + Yogue virus‡	NT	NT	NT	NT	0/3	1/272 (0.4)	1/275 (0.4)	1
F	NT	NT	NT	NT	NT	1/126	1/126	1
Adult	NT	NT	NT	NT	NT	1/46	1/46	1

Infected bats	Uganda: Python Cave	Uganda: Kasungwa Cave	Uganda: Kitaka Mine	Uganda: Total no.	Sierra Leone: Tailu Village	Sierra Leone: Kasewe Cave	Sierra Leone: Total no.	Grand total
Juvenile	NT	NT	NT	NT	NT	0/80	0/80	0
M	NT	NT	NT	NT	0/3	0/145	0/148	0
Adult	NT	NT	NT	NT	0/1	0/61	0/62	0
Juvenile	NT	NT	NT	NT	0/2	0/84	0/86	0
Sosuga + Kasokero virus†	1/558 (0.2)	NT	0/384	1/942 (0.1)	NT	NT	NT	1
F	1/218	NT	0/194	1/412	NT	NT	NT	1
Adult	1/125	NT	0/99	1/224	NT	NT	NT	1
Juvenile	0/93	NT	0/95	0/188	NT	NT	NT	0
M	0/339	NT	0/190	0/529	NT	NT	NT	0
Adult	0/209	NT	0/124	0/333	NT	NT	NT	0
Juvenile	0/130	NT	0/66	0/196	NT	NT	NT	0
Sosuga + Yogue virus‡	NT	NT	NT	NT	0/3	1/272 (0.4)	1/275 (0.4)	1
F	NT	NT	NT	NT	NT	1/126	1/126	1
Adult	NT	NT	NT	NT	NT	1/46	1/46	1
Juvenile	NT	NT	NT	NT	NT	0/80	0/80	0
M	NT	NT	NT	NT	0/3	0/145	0/148	0
Adult	NT	NT	NT	NT	0/1	0/61	0/62	0
Juvenile	NT	NT	NT	NT	0/2	0/84	0/86	0
Marburg + Sosuga + Yougue‡	NT	NT	NT	NT	0/3	1/272 (0.4)	1/275 (0.4)	1
F	NT	NT	NT	NT	NT	1/126	1/126	1
Adult	NT	NT	NT	NT	NT	1/46	1/46	1
Juvenile	NT	NT	NT	NT	NT	0/80	0/80	0
M	NT	NT	NT	NT	0/3	0/145	0/148	0
Adult	NT	NT	NT	NT	0/1	0/61	0/62	0
Juvenile	NT	NT	NT	NT	0/2	0/84	0/86	0

*Values are no. infected/total no. tested (%). Infections are listed by population demographics (sex and age) for each roost site. NT, not tested.
†Number positive out of 942 tested (Python Cave, n = 558; Kitaka Mine, n = 384). Python Cave bat no. 42 (adult female) was co-infected with Sosuga and Kasokero viruses.
‡Number positive out of 275 tested (Tailu Village, n = 3; Kasewe Cave, n = 272). Marburg + Sosuga + Yougue virus, Sosuga + Yogue virus, and Maburg + Yogue virus co-infections were detected in bat no. 1532 (adult female) from Sierra Leone.

Reference

16. Mungall BA, Middleton D, Crameri G, Halpin K, Bingham J, Eaton BT, et al. Vertical transmission and fetal replication of Nipah virus in an experimentally infected cat. J Infect Dis. 2007;196:812–6. PubMed https://doi.org/10.1086/520818
